# Interferon and TLR genes, but not endogenous bornavirus-like elements, limit BoDV1 replication after intracerebral infection

**DOI:** 10.1371/journal.ppat.1013165

**Published:** 2025-05-09

**Authors:** Rie Koide, Takaya Abe, Taichi Harimoto, Anselmo Jiro Kamada, Yuka Saito, Matteo Guerrini, Asami Fujii, Erica Parrish, Masayuki Horie, Hiroshi Kiyonari, Kazuhiko Yamamoto, Keizo Tomonaga, Nicholas F Parrish

**Affiliations:** 1 Genome Immunobiology RIKEN Hakubi Research Team, RIKEN Cluster for Pioneering Research and RIKEN Center for Integrative Medical Sciences, Yokohama, Japan; 2 Laboratory for Animal Resources and Genetic Engineering, RIKEN Center for Biosystems Dynamics Research, Kobe, Japan; 3 Department of Biology, University of Oxford, Oxford, United Kingdom; 4 Laboratory for Autoimmune Diseases, RIKEN Center for Integrative Medical Sciences, Yokohama, Kanagawa, Japan; 5 Graduate School of Veterinary Science, Osaka Metropolitan University, Osaka, Japan; 6 Osaka International Research Center for Infectious Diseases, Osaka Metropolitan University, Osaka, Japan; 7 Laboratory of RNA Viruses, Department of Virus Research, Institute for Frontier Life and Medical Sciences, Kyoto University, Kyoto, Japan; 8 Laboratory of RNA Viruses, Department of Mammalian Regulatory Network, Graduate School of Biostudies, Kyoto University, Kyoto, Japan; 9 Department of Molecular Virology, Graduate School of Medicine, Kyoto University, Kyoto, Japan; The Ohio State University, UNITED STATES OF AMERICA

## Abstract

Borna disease virus 1 (BoDV1) is a disease-causing agent in some livestock and, as has recently been shown, in humans. What constitutes a protective immune response to BoDV1 is unclear. Previous studies found that endogenous bornavirus-like nucleoprotein elements (EBLNs) present in mammalian genomes produce piRNAs antisense to BoDV1 nucleoprotein mRNAs. As a known function of piRNAs is to restrict transposons via RNA interference, it has been hypothesized that EBLN-derived piRNAs may restrict BoDV1. Here we used EBLN knockout (KO) and other KO mice to test genetic factors potentially involved in antiviral immunity to BoDV1. In previous reports, BoDV1 replication was higher in mice deficient in interferon gamma, and we confirmed a role for this cytokine in BoDV1 restriction at 12 weeks post infection using mice lacking its receptor. We show that BoDV1 replicates to higher levels in the brain of mice without Toll-like receptor 7 (TLR7), suggesting a role for this innate immune receptor in BoDV1 immunity. In contrast, mice lacking piRNA-producing EBLNs were no more susceptible to BoDV1 infection than wild-type under the infection conditions used here. We thus expand the genetic evidence implicating specific conventional immune pathways in BoDV1 control and conclude that EBLN-derived piRNA-guided antiviral silencing, if it occurs, is relatively less impactful in intracerebral infection of neonates.

## Author summary

The study examined the immune response to Borna disease virus 1 (BoDV1), which infects both animals and humans. Previous studies suggest that endogenous bornavirus-like nucleoprotein elements (EBLNs) within mammalian genomes might contribute to antiviral immunity against BoDV1 through a novel mechanism involving piRNA silencing. To dissect the potential role of EBLNs in BoDV1 immunity at a genetic level, we engineered EBLN knockout (KO) mice lacking all three piRNA-producing EBLNs. These mice, along with other KO mice lacking specific immune factors, were injected intracerebrally with BoDV1. The study confirmed that conventional immune pathways involving interferon gamma and Toll-like receptor 7 (TLR7) are more critical for controlling BoDV1 infection compared to the proposed mechanism involving EBLN-derived piRNAs, at least in the context of brain infections in newborn mice. These findings suggest that, while EBLNs might play a role in different scenarios, conventional immune pathways are likely the primary defense against BoDV1 infection, particularly in newborn mouse brains. Further research is needed to determine the significance of EBLNs in antiviral immunity.

## Introduction

Borna disease virus 1 (BoDV1) is a negative-stranded RNA virus in the family *Bornaviridae*. BoDV1 establishes a persistent infection in the central nervous system (CNS) and leads to neurological diseases in horses, sheep, and other mammalian species [1]. The bicolored white-toothed shrew, in which BoDV1 may replicate asymptomatically, is a known reservoir species for BoDV1 [2]. Although human BoDV1 infections remained controversial for decades, BoDV1 was shown to cause fatal encephalitis in humans after solid organ transplantation in 2018 [3]. Subsequently, more than 40 sporadic human BoDV1 encephalitis cases have been reported [4–9], raising awareness of the zoonotic risk posed by BoDV1.

Rodent models have primarily been used thus far to characterize the immune responses relevant to BoDV1 infection in the CNS. The pathogenic effects of BoDV1 infection in rats are influenced by multiple factors. For instance, different BoDV1 strains exhibit varying degrees of pathogenicity [10]. Additionally, genetic background is crucial in determining susceptibility; Lewis rats, but not Sprague-Dawley (SD) rats, generally develop neural abnormalities and hippocampal dysfunction following BoDV1 infection [11]. Similarly, in mice, different strains exhibit different degrees of pathology and behavioral changes after BoDV1, despite robust viral replication in the brain [12,13]. Furthermore, the animal's age at the time of infection significantly impacts disease progression. While experimental infection of adult C57BL/6 (WT) mice often takes an asymptomatic course, BoDV1 infection of newborn mice can result in neurological disease [13]. The immunopathogenesis of BoDV1 infection has mainly been attributed to T cell-based immune responses [14,15]. BoDV1 infection of newborn C57BL/6 and MRL mice lacking cytotoxic CD8 T cells does not usually result in neurological disease [13]. On the other hand, interferon gamma (IFNg) secreted by CD8 T cells was shown to mediate noncytolytic clearance of BoDV1 from hippocampal neurons, and *Ifng* KO mice show increased virus replication *in vivo* [16]. Thus, CD8 T cells and IFNg appear to play crucial roles in controlling BoDV1 infection, but the underlying mechanisms are still incompletely understood. BoDV1 has been proposed to limit activation of RNA-sensing innate immune receptors including MAVS [17]. Whether Toll-like receptor 7 (TLR7), which plays an important role in susceptibility to other neurotropic RNA viruses [18–20], is involved in BoDV1 control has not been tested.

Besides conventional immune mechanisms, it has been proposed that endogenous bornavirus-like nucleoprotein elements (EBLNs), sequences homologous to BoDV1 nucleoprotein gene in mammalian genomes, could contribute to protection against BoDV1 [21,22]. We previously showed that rodent and primate EBLNs are transcribed and processed into piRNAs antisense relative to BoDV1 nucleoprotein mRNAs in a pattern unexpected to arise via neutral evolution [23]. Because piRNAs are known to guide RNA interference against transposons, we hypothesized that EBLN-derived piRNAs may function as an immune defense mechanism against BoDV1 [23,24]. Antiviral activity of piRNAs was recently shown in mosquitoes [25], but has not been demonstrated in mammals.

In this study, we established a neonatal mouse model of BoDV1 infection to query the genetic constituents of BoDV1 immunity, including via a potential novel piRNA silencing mechanism. Using CRISPR/Cas genome engineering and breeding, we engineered EBLN knockout (KO) mice lacking all three piRNA-producing EBLNs. While the mouse brain does not express all factors used for piRNA biogenesis in the germline [26], transcription of pachytene piRNA clusters [27] and functional Piwi proteins [28–30] have been reported in this context. Therefore, the model established here enables us to address whether EBLNs are involved in antiviral immunity against BoDV1.

## Results

### Validation of assays to quantify viral RNA copies and titers

We used a recombinant BoDV1 carrying GFP reporter (rBoDV P/M-GFP) [31] for intracerebral injection in mice. To assess the infectivity and stability of rBoDV P/M-GFP, we first compared the replication kinetics of BoDV WT and rBoDV P/M-GFP on Vero cells. Early replication kinetics of rBoDV P/M-GFP in Vero cells were comparable to those of BoDV WT. After an eclipse phase of about 2 days, the percentage of infected cells began to increase exponentially, and viral spread to neighboring cells was observed from 3.5 days post inoculation (dpi) (S1A Fig). Thus, we used a 3 day incubation period for subsequent titration experiments. At 3 dpi, there was no significant difference between BoDV WT and rBoDV P/M-GFP as measured by the percentage of infected cells and the number of fluorescent foci, and at 3 days these are linearly related (S1B Fig). To ensure accurate measurement of virus in the presence of a complex biological mixture, a spike-in assay was performed using serially diluted virus stock spiked into uninfected homogenized brain tissue. There was a linear relationship between titers of input virus and viral RNA copies in spike-in solutions (S1C and S1D Fig).

### A murine model of BoDV1 infection

**Fig 1:**
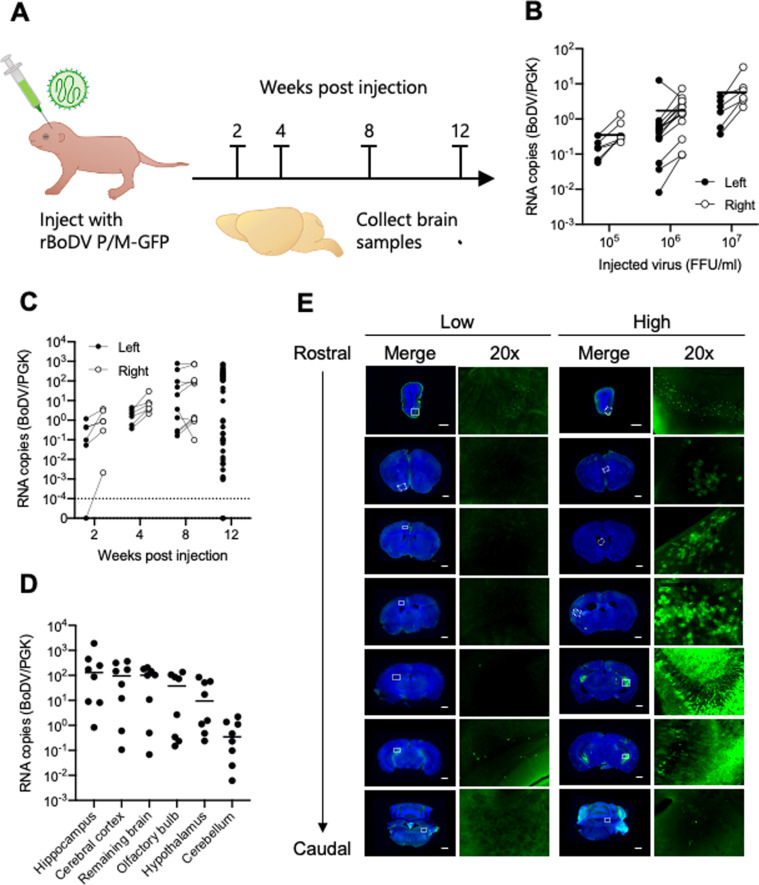
Establishment of a murine model of BoDV1 infection using C57BL/6J mice and rBoDV P/M-GFP. (A) Schematic diagram of the experimental infection. Neonatal C57BL/6J mice were injected in the right lateral ventricle with rBoDV P/M-GFP. Brain tissues were collected at certain points from 2 to 12 wpi for virological analysis. (B) Animals were injected with different titers of virus (FFU/ml), and viral RNA copies in the left and right brains were quantified after 4 wpi. (C) Kinetics of rBoDV P/M-GFP in left and right brains. (D) Viral RNA copies in different regions of brains (olfactory bulb, cerebral cortex, hippocampus, hypothalamus, cerebellum, remaining brain) at 12 wpi. (E) Survey of rBoDV P/M-GFP in coronal brain sections at 12 wpi. Representative images of the brains from animals with low or high virus replication are shown. Brain sections were counterstained with DAPI (Nacalai Tesque). Scale bar, 1 mm.

We next established a neonatal mouse model of BoDV1 infection using rBoDV P/M-GFP. A schematic of the experimental design is shown in Fig 1A. Although a previous study showed that BoDV1 experimental infection using brain-derived stock results in encephalitis in approximately 15% of C57BL/6J mice [13], in our model mice remained asymptomatic and showed no evidence of neurological dysfunction, even when closely monitored for provoked postural differences and weight loss, which are common clinical signs of Borna disease [13]. Thus, we assessed BoDV replication by viral RNA quantification and histological analysis. To determine how different viral inoculum affects viral replication, groups of mice were injected with different titers of rBoDV P/M-GFP. 4 weeks post injection (wpi), viral RNA copies in brain had increased in a titer-dependent manner (Fig 1B). Viral RNA loads were most often higher on the right brain, the side of injection, but virus also spread to the left brain, indicative of virus trafficking and most consistent with active virus replication. A time-course analysis showed that brain viral RNA load rises over 4 weeks in all injected animals, then declines in some animals. This suggests differential control of virus infection (Fig 1C). We dissected various regions of infected brains and compared the viral RNA loads at 12 wpi. Viral loads were highest in the hippocampus and lowest in the cerebellum (Fig 1D). We then performed histological analysis and assayed for rBoDV P/M-GFP in coronal brain sections. Representative images of brains from animals with low or high virus replication are shown in Fig 1E. Consistent with our qPCR results, we observed higher GFP expression in the hippocampus compared with other regions, and only weak GFP signal was observed in the cerebellum.

### Tropism of rBoDV P/M-GFP in different brain cell types

**Fig 2:**
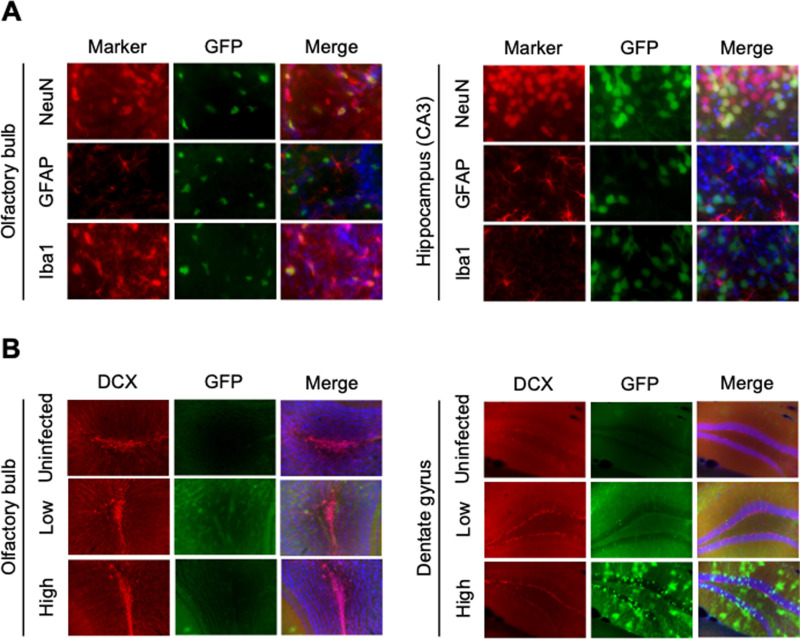
Histological analysis of mouse brains infected with rBoDV P/M-GFP. (A) Detection of rBoDV P/M-GFP in different brain cell types in olfactory bulb and CA3 region of hippocampus. Brain sections of animals injected with rBoDV P/M-GFP were stained with antibodies to microglia (Iba-1), astrocytes (GFAP) and neurons (NeuN). Brain cell specific markers are labeled in red and counterstained with DAPI (Nacalai Tesque). (B) Absence of cytoarchitectural change in olfactory bulb and dentate gyrus of BoDV-infected animals. Uninfected animals and animals infected with rBoDV P/M-GFP at 12 wpi were stained for DCX (red). Low, animal showing low BoDV1 replication; High, animal showing high BoDV1 replication. There was no reduction in DCX-positive granule cells, regardless of virus replication levels.

We next investigated different brain cell types that are susceptible to rBoDV P/M-GFP by conducting histological analysis with antibodies to cell type specific markers of neurons (neuronal nuclear antigen [NeuN]), astrocytes (Glial fibrillary acidic protein [GFAP]) and microglia (Ionized calcium binding adaptor molecule 1 [Iba-1]). We focused on two brain regions, the hippocampus and olfactory bulb, where we consistently observed high viral replication. In the hippocampus, GFP expression appeared restricted to neurons, while we observed infection of neurons and microglia in the olfactory bulb (Fig 2A). We also stained brain slices with antibodies against doublecortin (DCX), a specific marker of neural precursor cells (NPCs), given reports of Piwi protein expression in these cells [32]. However, we did not detect GFP expression in DCX-positive cells, including in brains with high BoDV1 replication (Fig 2B).

### Validation of our model with known immune defects

**Fig 3:**
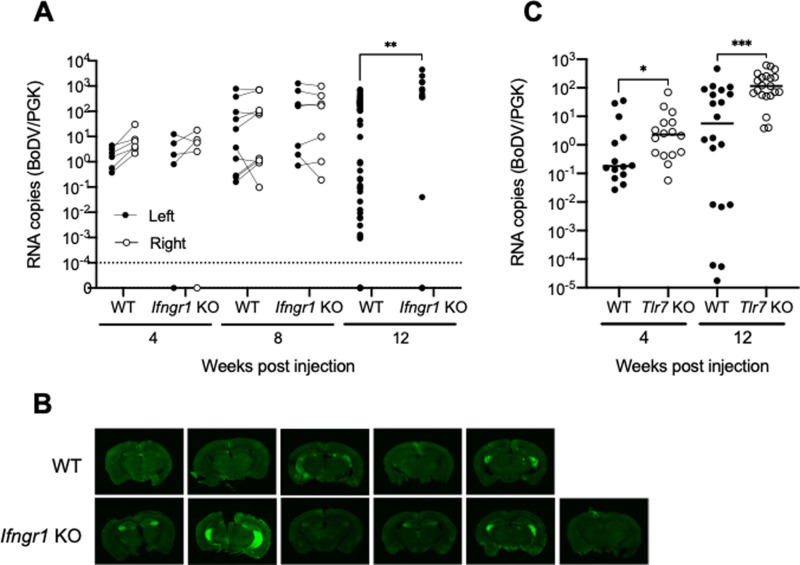
Effect of *Ifngr1* KO and *Tlr7* KO on rBoDV P/M-GFP infection. (A) Viral RNA copies in WT and *Ifngr1* KO at the indicated time points. When there was no amplification in the samples, we defined it as zero RNA copies. (B) Representative images of coronal brain sections at 12 wpi from 5 different animals each for WT and *Ifngr1* KO mice. (C) Viral RNA copies in WT and *Tlr7* KO at the indicated time points.

We next applied these experimental techniques to mice lacking components of the immune system with a high prior probability to demonstrate a phenotype in anti-BoDV immunity. Transgenic mice lacking interferon gamma receptor 1 chain receptor (*Ifngr1* KO) or TLR7 signaling (*Tlr7* KO) were infected as above. By 12 wpi, BoDV1 replication in brains of *Ifngr1* KO mice exceeded that of WT mice (Fig 3A), confirming the previous finding that IFNg is essential for immune control of BoDV1 infection using an orthogonal mutation. We confirmed GFP expression in the coronal brain sections, and GFP expression in *Ifngr1* mice appeared to be higher when compared to those in WT mice (Fig 3B). Viral RNA copies began to increase in some *Tlr7* KO mouse brains at 4 wpi compared to WT mice (Fig 3C). By 12 wpi, the impact of TLR7 deficiency became more pronounced, with significantly increased viral RNA copies in *Tlr7* KO mouse brains compared to WT mice, and very few mice suppressing replication to low levels (Fig 3C). To the best of our knowledge, this is the first report that TLR7 plays a role in host immunity against BoDV1 infection. These experiments demonstrated that the techniques used here, despite significant intra-individual variation, are able of detect genetic effects on BoDV1 immunity, and that by 12 weeks two conventional immune deficiencies manifest such an effect.

### Generation of mice lacking all piRNA-producing EBLNs by genome editing

**Fig 4:**
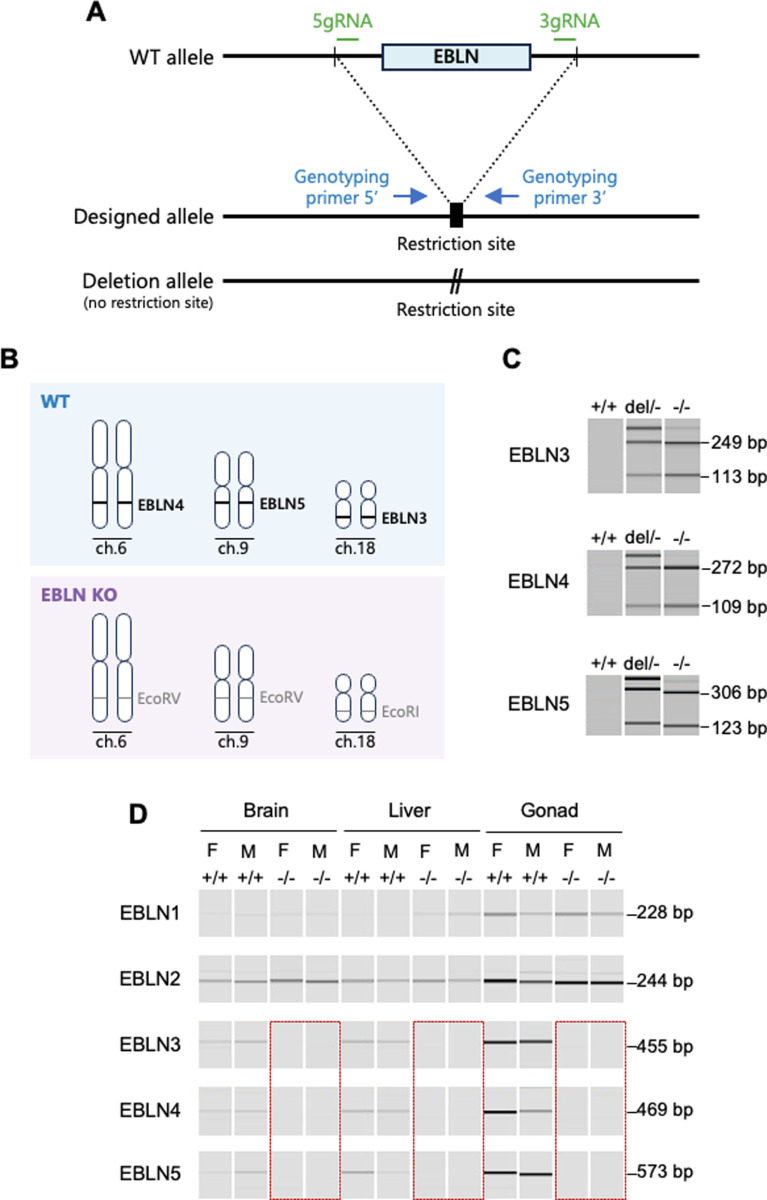
CRISPR/Cas9-mediated generation of EBLN KO mouse model. (A) Schematic diagram of the strategy used to generate EBLN KO mice. The binding sites for gRNAs are shown in green, and the target EBLN sequence is indicated with a light blue box. The designed allele has a restriction site marked with a black box that allows us to distinguish from deletion alleles, which have other CRISPR-induced mutations. Blue arrows represent the genotyping primers. (B) A total of five EBLNs are present in the mouse genome. Three of the five EBLNs (EBLN3, EBLN4 and EBLN5) located on chromosomes 18, 6 and 9 of the mouse genome, respectively, are known to produce piRNAs antisense to the BoDV1 N mRNA sequence. These were removed from the mouse genome using CRISPR/Cas9. EBLN KO mouse lacks all EBLN3, EBLN4 and EBLN5, and restriction sites (EcoRV or EcoRI) were introduced at the targeted KO sites. (C) A representative genotyping result for WT (+/+), heterozygous KO with the deletion allele (del/-), and homozygous KO (-/-) mice. PCR followed by a restriction digest was used to distinguish the designed allele and deletion alleles. The amplicon size is shown on the right. (D) EBLN expression in brains, livers and gonads in WT (+/+) and EBLN KO (-/-) mice. Absence of EBLN3, EBLN4 and EBLN5 expression was confirmed in EBLN KO (-/-) mice. Gender of each mouse is shown below each line. F, female; M, male. The amplicon size is shown on the right.

In the mouse, piRNAs are generated from three of five EBLNs (EBLN3, EBLN4, EBLN5), which are present within or close to piRNA clusters [23]. To investigate the role of EBLN-derived piRNAs, we aimed to generate EBLN KO mice lacking all three EBLNs that produce piRNAs. As shown in Fig 4A, two guide RNA (gRNA) target sites were designed flanking each EBLN sequence, and a new restriction site (EcoRV or EcoRI) was introduced to facilitate mutant screening. CRISPR/Cas9 with three pairs of gRNAs was used to target EBLN3, EBLN4, and EBLN5 located on chromosomes 18, 6, and 9, and an EcoRI or EcoRV site was inserted at each targeted locus (Fig 4B). Genotypes of the resulting pups were determined by PCR followed by restriction digestion (Fig 4C). While we attempted to generate mice lacking all three EBLNs, none of the mice at the first generation had all three knockouts of EBLNs. Thus, we bred these mice together to obtain mice lacking all EBLN3, EBLN4 and EBLN5, which we designated as EBLN KO mice. Expression of EBLNs in control mice, and absence of expression in EBLN KO mice, was consistent with expectations (Fig 4D).

### Comparison of BoDV1 outcomes in WT, EBLN het and EBLN KO animals

**Fig 5:**
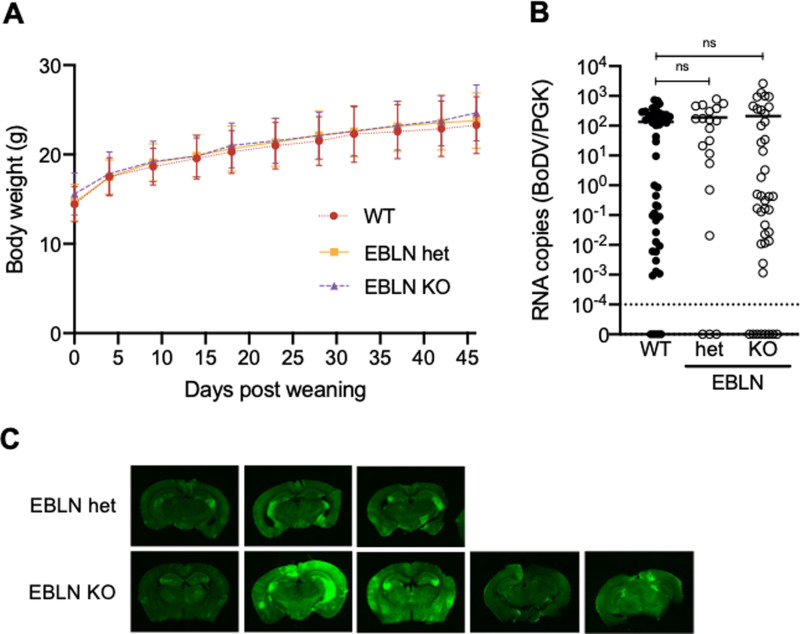
Comparison of susceptibility to BoDV1 infection in WT, EBLN heterozygous (het) and EBLN KO animals. (A) Body weight in WT, EBLN het and EBLN KO animals injected with rBoDV P/M-GFP after weaning. There were no significant differences in weight among these animals. (B) Comparison of viral RNA copies in WT, EBLN het and EBLN KO animals at 12 wpi. Viral RNA copies in EBLN KO animals were not significantly different compared to those in EBLN het or WT animals. (C) Representative images of coronal brain sections from 3 different animals each for EBLN het and EBLN KO mice.

Finally, we performed BoDV1 experimental infection by injecting WT, EBLN KO and EBLN heterozygous neonatal mice with rBoDV P/M-GFP. We monitored the body weight of injected animals after weaning, and there were no significant differences in weight among these animals (Fig 5A). Thus, BoDV replication was assessed by RT-qPCR and histology. We compared the whole-brain viral loads in WT, EBLN het and EBLN KO animals at 12 wpi. No significant difference was observed in whole-brain viral loads of EBLN KO mice compared to EBLN het and WT controls (Fig 5B). An exploratory survey of GFP expression in coronal brain sections revealed that GFP expression in the hippocampus appeared higher in some highly-infected EBLN KO mice compared with EBLN het and WT animals (Figs 5C and 3B), yet the number of animals analyzed in this manner was low. Heterogeneity of infection outcome in this system and the resulting low statistical power currently preclude experiments to investigate whether this phenotype is genetically determined.

## Discussion

Bornavirus-like endogenous viral elements integrated in eukaryote genomes act as a record of long-term interactions between these viruses and diverse hosts [33]. Despite the importance of BoDV1 as a zoonotic human pathogen, our understanding of the immune factors involved in its control, including the EBLNs that are potentially such a factor, remains rudimentary. A previous study demonstrated that an EBLN in the thirteen-lined ground squirrel genome inhibits BoDV1 infection as a dominant negative form of viral protein when overexpressed in cell lines [34]. As an alternative hypothetical mechanism, we proposed that small RNAs produced from some rodent and primate EBLNs may target BoDV1 sequences and act as an antiviral factor against BoDV1 infection [23]. This is the first study to test if such EBLNs are involved in anti-BoDV1 immunity by experimental infection of mice.

Deleting the three piRNA-producing EBLNs did not affect the whole-brain viral loads in infected animals at 12 wpi. If EBLN-derived piRNAs have an antiviral activity against BoDV1 infection, their protective effect is less than that of IFNg or TLR7 under these conditions. Our assays are of limited granularity and resolution; we cannot exclude the possibility that we would observe differences in a specific tissue or at different time points or under different infection conditions. Because piRNAs are abundant in germ cells [35–38], piRNA-mediated effects would be predicted to be highest in gonads, which may also harbor BoDV1 replication. In mosquitoes, excision of an EVE by genome editing allowed a higher increase in replication of a cognate virus in ovaries compared to heads [25], suggesting a stronger effect of EVE-mediated antiviral immunity in germ cells. piRNA expression has been reported in hippocampal neural progenitor cells (NPCs) [32]. Thus, it remains reasonable to hypothesize that EBLN-derived piRNAs could play a role in germ cells or specific brain cells or regions despite these observations using whole brains.

There was considerable variability in rBoDV P/M-GFP replication in mouse brains at 12 wpi. As this variability was essentially lost in *Tlr7* KO mice, and decreased in *Ifngr1* KO mice, we suspect that variation in immune response is a large factor. While our study provides valuable insights, the current sample size may limit our ability to detect small effects. Future studies with larger groups of animals could increase statistical power and help to further investigate the sources of this variability. *Tlr7* KO mice generate poor neutralizing antibody responses to various viruses, raising this as one candidate immune control mechanism yet unexplored for BoDV1 [39–41]. Our work more than doubles the number of genetic deficiencies known to impact BoDV1 control, highlighting this as an important area for future experiments. Some of the variability of viral RNA loads may have resulted from technical causes; here we injected all animals by hand. However, as we more recently use a stereotactic injector yet have continued to observe similar variation, improving this technical aspect alone is unlikely to substantially improve our ability to detect smaller effects.

Another important caveat to our hypothesis is the degree of mismatch between EBLN sequences and the BoDV1 strain used here. For example, the nucleotide identity between EBLN 5 sequence and modern BoDV1 is about 50% across the entire EBLN sequence, although greater identity is preserved in short regions. Because the base pairing between piRNAs and target sequences is important for piRNA recognition [42–43], mismatches between EBLN-derived RNAs and BoDV1 mRNA could prevent efficient silencing that our experimental system would have otherwise detected. Thus, a mouse model with an "artificially endogenized" sequence of BoDV1 would be useful to test whether integration of an EBLN-like sequence could have, at some point in the course of evolution (e.g., against a cognate virus shortly after integration), functioned in the role hypothesized here.

We used C57BL/6 mice as they enabled the use of existing mutants as well as CRISPR-mediated generation of additional mutants. While this model offers advantages for genetic manipulation, its inherent low susceptibility to BoDV1 and lack of typical T cell-mediated immunopathology limited our ability to fully replicate the complex immune responses observed in more susceptible hosts. Thus, our results should be interpreted in this context, which differs in key ways from other models. For experimental infection, we utilized a GFP reporter virus expanded in cell culture, rather than the brain passage-derived stocks commonly employed in previous BoDV1 animal models, to improve reproducibility. Despite its potential advantages, the rBoDV P/M-GFP infection exhibited atypical characteristics, including the absence of clinical disease and a decrease in viral load at 12 wpi in some animals. The recombinant nature of the virus, the cell culture conditions used for propagation, and the integration of the GFP tag itself are all potential contributing factors to these altered responses. Specifically, presence in microglia in the olfactory bulbs, which we favor to represent infection rather than phagocytosis on the basis of the distribution of the GFP signal, is rarely observed in other model systems. These limitations underscore the challenges of fully capturing BoDV1 pathogenesis in this model.

In this study, we investigated the potential antiviral role of EBLNs in BoDV1 infection. We established an animal model of BoDV1 infection by intracerebrally injecting C57BL/6 mice with recombinant BoDV1 carrying GFP reporter. Using genome-engineered mouse models, we confirmed the previously reported role of interferon gamma and identified a novel role for TLR7 in controlling BoDV1 infection. Despite an assay system capable of resolving the impact of these immune deficits, we found no evidence of inhibitory effects of EBLNs on BoDV1 infection within the brain. While our current findings do not support a direct antiviral role for EBLNs in BoDV1 infection, our established animal model provides a valuable platform for future research. Notable with respect to this hypothesis, a role for virus-derived antisense piRNAs in reducing expression of koala retrovirus A envelope glycoprotein expression in somatic tissues was shown while this work was under review [47]. By manipulating mouse strains and viral genotypes or examining specific tissues and time points, we may uncover novel functions of EBLNs in BoDV1 infection.

## Materials and methods

### Ethics statement

Approval for animal experiments was obtained from the Institutional Animal Care and Use Committee of RIKEN Kobe (approval number: A2001-03) and Yokohama (approval number: AEY2024–022) Branches and was taken out in accordance with international standards.

### Cells

Vero cells (ATCC# CCL-81) and Vero cells stably expressing rBoDV P/M-GFP were maintained in Dulbecco’s modified Eagle’s medium (DMEM) (Nacalai Tesque, Kyoto, Japan) supplemented with 2% fetal bovine serum (FBS) and penicillin-streptomycin.

### Viruses

Virus stock of cell-associated rBoDV P/M-GFP [31] was prepared from sonicated cell lysates of Vero cells persistently infected with rBoDV P/M-GFP. The cells were detached by trypsin and suspended in Opti-MEM (Nacalai Tesque). The cell suspension was then sonicated for 2.5 min (5 cycles, 30 s each) using a Bioruptor sonicator (Sonic Bio, Kanagawa, Japan) and centrifuged at 1,200 g for 25 min. The supernatant was stored as a virus stock at -80°C until use.

### Virus titration

Virus titration was carried out in 96-well plates. Vero cells were seeded one day earlier at 5x10^3 cells/well, and serial dilutions of cell-free virus stocks were added to each well. After 3 days of incubation at 37°C, the cells were fixed, and infectious units were determined by counting GFP fluorescence or using the fluorescent antibody technique described below. Fluorescent foci were counted for an appropriate dilution to obtain the infectious unit per ml of the inoculum.

### Indirect immunofluorescence assay

Virus-inoculated cells were fixed with 4% paraformaldehyde at RT for 15 min and then permeabilized by PBS with 0.25% Triton-X-100 for 5 min. After blocking with 1% bovine serum albumin with 0.1% Tween-20 in PBS at RT for 30 min, the cells were incubated with the primary antibody, rabbit polyclonal anti-BoDV1 N HB01 [44] for 1 h at RT at a 1:2,000 dilution. Subsequently, the cells were washed with PBS and incubated with the secondary antibody, goat anti-rabbit IgG conjugated with Alexa Fluor 555 (ab150078; Abcam, Cambridge, UK) at a 1:2,000 dilution for 1 h at RT. The cells were counterstained with 4′,6′-diamidino-2-phenylindole (DAPI) (Nacalai Tesque). After washing with PBS, the cells were observed using a fluorescence microscope BZ-X700 (Keyence, Osaka, Japan).

### Replication kinetics

To measure virus replication, Vero cells were seeded at 2x10^3 cells/well one day prior to infection with BoDV1 at a multiplicity of infection (MOI) of 1 or 5 in a 96 multi-well plate. After 2 h at RT for adsorption and 30 min at 37°C for penetration, the cells were washed three times with PBS and then cultured in fresh DMEM supplemented with 10% FBS, penicillin and streptomycin. At the time points indicated in S1 Fig, virus propagation was detected by GFP fluorescence or indirect immunofluorescence assay as described above. The percentage of infected cells was calculated by dividing the number of cells positive for fluorescent foci by the total number of cells using a fluorescence microscope BZ-X700.

### Spike-in assay using uninfected brain

Serially diluted rBoDV P/M-GFP virus stock was mixed with uninfected whole-brain tissue lysate in varying ratios. RNA was extracted from each sample with serial dilution of virus and subjected to RT-qPCR to measure the viral RNA loads. RNA extraction and RT-qPCR were performed as described below.

### Animal experiments

C57BL/6J mice were obtained from CLEA, Japan (Shizuoka, Japan). *Ifngr1* KO mice were kindly provided by Dr. Shigeo Koyasu (RIKEN Center for Integrative Medical Sciences, Kanagawa, Japan). Neonates were injected in the right lateral ventricle with 10^4 FFU of rBoDV P/M-GFP under hypothermic anesthesia [45]. After weaning, body weight was measured three times a week. For necropsy, animals were euthanized by cervical dislocation, and tissues including brains, livers and gonads were collected immediately for further analysis.

### Generation of EBLN KO mice

EBLN KO (EBLN3 KO, EBLN4 KO and EBLN5 KO) mice (CDB Accession No.: CDB0086E: https://large.riken.jp/distribution/mutant-list.html) were generated by single-strand oligodeoxynucleotide (ssODN)-mediated knock-in with CRISPR/Cas9-mediated genome editing using C57BL/6N zygotes. For electroporation, the zygotes were transferred into Opti-MEM I (ThermoFisher Scientific) containing six crRNA (7.5ng/μl), tracrRNA (100ng/μl), three ssODN containing an EcoRI or an EcoRV restriction enzyme recognition site (400ng/μl) and Cas9 protein (250ng/μl) (Thermo Fihser Scientific). CUY21 EDIT II and LF501PT1–10 platinum plate electro code (BEX Co. Ltd.) were used and electroporation conditions were 30 V (3 msec ON + 97 msec OFF) x 7 times [46]. After electroporation, the zygotes were transferred into pseudopregnant ICR female mice. From 116 zygotes, 9 founder mice were obtained and genotyped by PCR. The crRNA, tracrRNA and ssODN are listed in S1 Table.

### Genotyping of EBLN KO mice

Genotyping of EBLN KO mice was performed by PCR followed by restriction enzyme digestion. Standard phenol-chloroform extraction method was used to extract genomic DNA from mouse tails. Following DNA extraction, PCR was performed using TaKaRa Taq (TaKaRa Bio, Shiga, Japan) according to the manufacturer’s instructions. Conditions for PCR amplification were 1 cycle of pre-denaturation at 95°C for 5 min followed by 30 cycles of denaturation at 94°C for 30 s, annealing at 60°C (for EBLN3 and EBLN5) or 57°C (for EBLN4) for 30 s and extension at 72°C for 1 min and 1 cycle of post-extension at 72°C for 5 min. The primers used for genotyping PCR are listed in S2 Table. The PCR products were then digested by EcoRI (for EBLN3) or EcoRV (for EBLN4 and EBLN5) at 37°C overnight. The resulting digestion products were analyzed by MultiNA (Shimadzu, Kyoto, Japan) microchip electrophoresis system.

### RNA extraction and reverse transcription from mouse tissues

Harvested tissues including brains, livers and gonads were immediately stored in RNAprotect Tissue Reagent (QIAGEN, Hilden, Germany) and homogenized with Micro Smash MS-100R (TOMY MEDICO, Tokyo, Japan). Total RNA was extracted from homogenate using RNeasy Mini Kit (QIAGEN) according to the manufacturer’s instructions. cDNA was synthesized using Verso cDNA Synthesis Kit (Thermo Fisher Scientific, Waltham, MA, USA). For the RT reaction, 10 μl of reaction mixture containing 2 μl of 5X cDNA Synthesis Buffer, 1 μl of dNTP Mix (5 mM each), 0.5 μl of RT Enhancer, 0.5 μl of Verso Enzyme Mix, 0.5 μl of Anchored Oligo dT and 100 ng of total RNA was incubated at 50°C for 60 min, followed by inactivation step at 95°C for 2 min.

### Analysis of EBLN RNA expression by PCR

PCR was performed from cDNA template using Applied Biosystems GeneAmp PCR System 9700 (Thermo Fisher Scientific) with KOD One PCR Master Mix (Toyobo, Osaka, Japan) following the manufacturer’s instructions. Amplification conditions were 1 cycle of 98°C for 2 min and 35 cycles of 98°C for 10 s and 68°C for 5 s and 1 cycle of 72°C for 5 min. The primers used for PCR are presented in S2 Table.

### Determination of viral RNA copy number by qPCR

qPCR was performed with cDNA template and THUNDERBIRD Probe qPCR Mix (Toyobo, Osaka, Japan) using Applied Biosystems ViiA7 Real-Time PCR System (Thermo Fisher Scientific). Amplification conditions were 1 cycle of 95°C for 30 s and 40 cycles of 95°C for 5 s and 60°C for 30 s. The primers and probes used for qPCR are

shown in S2 Table. For determination of viral RNA copies, standard curves were generated by serial dilution of plasmids bearing the target sequences.

### Histology and immunohistochemistry

To prepare tissues, animals were perfused with 4% paraformaldehyde (PFA) in PBS (Nacalai Tesque) via cardiac puncture following cervical dislocation. Brains were dissected and post-fixed in 4% PFA overnight. Brain samples were then equilibrated in 20% sucrose overnight and stored at 4°C until use. A Leica VT1200S vibratome (Leica Biosystems, Wetzlar, Germany) was used to make coronal sections of 50 μm thickness. Selected sections were stained with DAPI (Nacalai Tesque) and mounted with Fluoromount-G (SouthernBiotech, Alabama, USA) on CREST-coated glass slides (Matsunami Glass, Osaka, Japan). GFP and DAPI images of entire brain sections were acquired using BZ-X700 fluorescence microscope (Keyence). For immunohistochemistry, sections were blocked with 2% BSA, 0.05% Tween-20, 0.1% Triton-X in PBS for 1 h, and incubated with desired primary antibodies diluted in blocking buffer for 48 h at a 1:1,000 dilution. Primary antibodies used were as follows: rabbit polyclonal anti-NeuN (ab104225), rabbit polyclonal anti-GFAP (ab7260), rabbit monoclonal anti-Iba1 (ab178846), and rabbit polyclonal anti-DCX (ab18723) (all from Abcam). Following incubation in primary antibodies, sections were washed with PBS and incubated overnight with goat anti-rabbit IgG conjugated with Alexa Fluor 555 (ab150078; Abcam) at a 1:1,000 dilution. Tissues were washed with PBS and counterstained with DAPI. After further washing with PBS, the sections were mounted in Fluoromount-G (SouthernBiotech). Immunostained sections were analyzed using a BZ-X700 fluorescence microscope (Keyence).

## Statistical analysis

Data were analyzed and visualized by using GraphPad Prism version 9 (GraphPad Software, La Jolla, CA, USA). Mann-Whitney test was used for comparison of viral RNA loads as determined by RT-PCR.

## Supporting information

S1 Fig Validation of titration and viral RNA quantification assays. (A) Replication kinetics of BoDV WT and rBoDV P/M-GFP on Vero cells. Vero cells were inoculated with BoDV WT and rBoDV P/M-GFP at a MOI of 1 and 5. Virus infection was monitored by GFP fluorescence or indirect immunofluorescence assay. (B) Estimation of percentage of cells infected based on MOI at 3 dpi. Percentage of infected cells was calculated by dividing the number of GFP positive cells by the total number of cells as determined using a BZ-X700 fluorescence microscope (Keyence). (C) Estimation of number of fluorescent foci based on MOI at 3 dpi. (D) Spike-in assay demonstrates a correlation between infectious titers and viral RNA copies.
(TIF)

S1 Table List of crRNAs, ssODNs, tracRNAs used to make EBLN KO mice. (PDF)

S2 Table List of primers and probes for genotyping, RNA expression and viral RNA quantification. (PDF)

S3 Table Raw data used to generate figures. (XLSX)
