# Supplementary figures and images for: Interferon and TLR genes, but not endogenous bornavirus-like elements, limit BoDV1 replication after intracerebral infection
Source: PLoS Pathog. 2025 May 9;21(5):e1013165. doi: 10.1371/journal.ppat.1013165 (PMC12112416; doi:10.1371/journal.ppat.1013165)

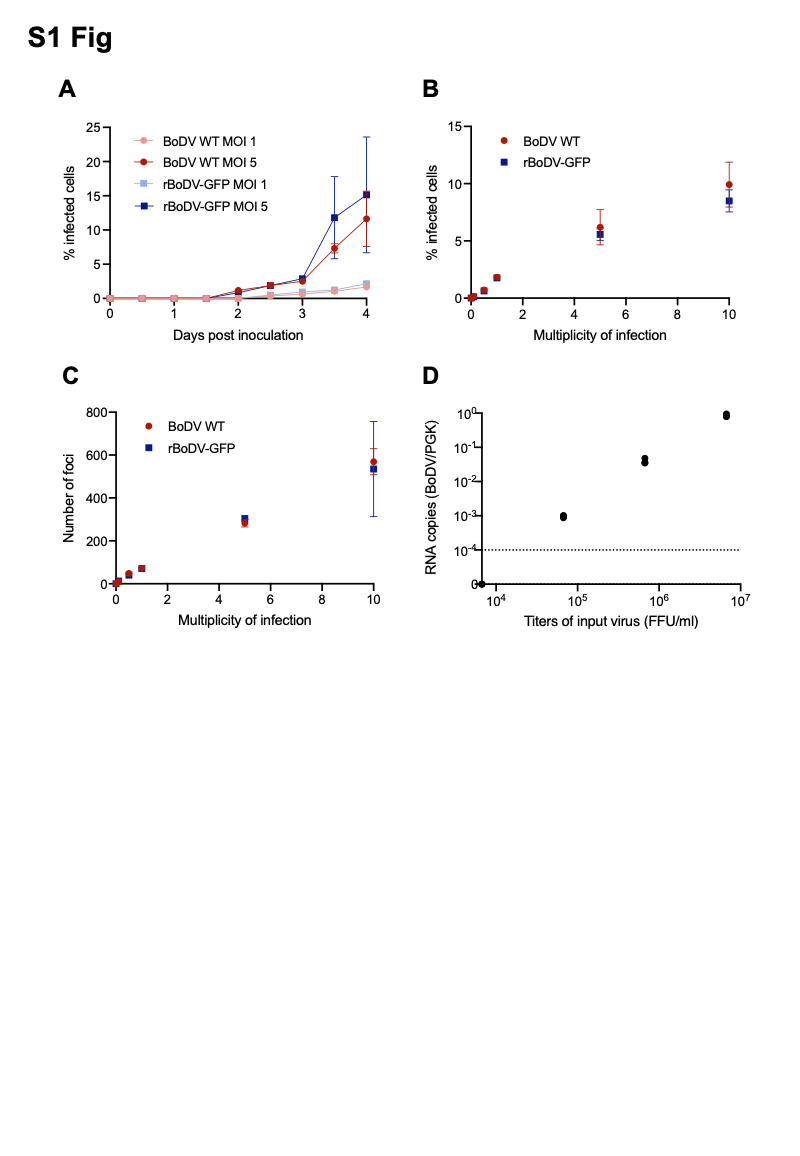

Supplement: S1 Fig — (A) Replication kinetics of BoDV WT and rBoDV P/M-GFP on Vero cells. Vero cells were inoculated with BoDV WT and rBoDV P/M-GFP at a MOI of 1 and 5. Virus infection was monitored by GFP fluorescence or indirect immunofluorescence assay. (B) Estimation of percentage of cells infected based on MOI at 3 dpi. Percentage of infected cells was calculated by dividing the number of GFP positive cells by the total number of cells as determined using a BZ-X700 fluorescence microscope (Keyence). (C) Estimation of number of fluorescent foci based on MOI at 3 dpi. (D) Spike-in assay demonstrates a correlation between infectious titers and viral RNA copies. (TIF) [file ppat.1013165.s001.tif]
