# Supplementary material for: Interferon and TLR genes, but not endogenous bornavirus-like elements, limit BoDV1 replication after intracerebral infection
Source: PLoS Pathog. 2025 May 9;21(5):e1013165. doi: 10.1371/journal.ppat.1013165 (PMC12112416; doi:10.1371/journal.ppat.1013165)
Supplement: S1 Table — (PDF) [file ppat.1013165.s002.pdf]

S1 Table.

Name	Sequence (5'-3')
EBLN3_5’-crRNA	GUG CAC CAU AAU GCC CAA GAG UUU UAG AGC UAU GCU GUU UUG
EBLN3_3’-crRNA	CAU CCC ACC AUG UCU CAA CCG UUU UAG AGC UAU GCU GUU UUG
EBLN3_ssODN	ATT CTT CTC CCT CAC CTT CCT GCA TGC TGG GAT TAC AGG TGT GCA CCA TAA TGC CCA GAA TTC TGA GAC ATG GTG GGA TGT TCT CTG GGT GTC TTT CTC CAT TGT TCT CTA CCT AAC TAT
EBLN4_5’-crRNA	AGA UGG CAA UGG UUG AGC CAG UUU UAG AGC UAU GCU GUU UUG
EBLN4_3’-crRNA	GCC AGC CAU AGC AAU UGC CCG UUU UAG AGC UAU GCU GUU UUG
EBLN4_ssODN	TGT CCT GAT ACT GTA ATG ACT ACC CAA GCG TCC CTG TTG AAG ATG GCA ATG GTT GAG GAT ATC CAA TTG CTA TGG CTG GCT GAA AGA CCT TAA CCA TGG AGC TTT TTC ATC TCT CCT ATG
EBLN5_5’-crRNA	CCU GUA GCU GAC CUG ACG AAG UUU UAG AGC UAU GCU GUU UUG
EBLN5_3’-crRNA	AGA GUG AGG UGA AGA GCG UUG UUU UAG AGC UAU GCU GUU UUG
EBLN5_ssODN	CTG TGG TTG AGC TTC TTG ATC ACA CAG CCA TCT TTT CCT TCC TGT AGC TGA CCT GAC GAT ATC GCT CTT CAC CTC ACT CTG AGG GCT TCT GAG AGA GAG GTT TGA GGG AAA CAG
tracrRNA	AAA CAG CAU AGC AAG UUA AAA UAA GGC UAG UCC GUU AUC AAC UUG AAA AAG UGG CAC CGA GUC GGU GCU
