# Supplementary material for: Interferon and TLR genes, but not endogenous bornavirus-like elements, limit BoDV1 replication after intracerebral infection
Source: PLoS Pathog. 2025 May 9;21(5):e1013165. doi: 10.1371/journal.ppat.1013165 (PMC12112416; doi:10.1371/journal.ppat.1013165)
Supplement: S2 Table — (PDF) [file ppat.1013165.s003.pdf]

S2 Table.

Name	Sequence (5'-3')
Genotyping	
EBLN3.gt_F	TAGTCCAGGCTAACCTGG
EBLN3.gt_R	GCACTCACAACACAAGCC
EBLN4.gt_F	CCACTAGGGCTGAGTAGG
EBLN4.gt_R	ACCTTCTCGCTGAGAAGC
EBLN5.gt_F	CTGACAGGCTTGCCTTGG
EBLN5.gt_R	TCAGCTAGGGGCTTGAGG
RNA expression	
EBLN1_F	TCCATGCACTGCTCCAAGTA
EBLN1_R	GGAAATCTGGCCATGCAGAG
EBLN2_F	CACACTGACACTGCAACACA
EBLN2_R	GGTATCTCAAAAGCGGCCTG
EBLN3_F	CTGAGACTGCACTGCTTCACAC
EBLN3_R	GTCACTTGCAACCTGCGGTAAG
EBLN4_F	TTGTGACTGGACCACCTGAGAAG
EBLN4_R	TTACTCCGGAAAAGTGCTGCTG
EBLN5_F	AAGCCCTCAGAGTGAGGTGAAG
EBLN5_R	TCTTTCACCTTTGGGGGTCAGC
Viral RNA quantification	
PGK_F	TGCACGCTTCAAAAGCGCACG
PGK_R	AAGTCCACCCTCATCACGACCC
PGK_probe	FAM-CCTCATCTC-ZEN-CGGGCCTTTCGACCTCA-3IABkFQ
BoDV_F	ATGCATTGACCCAACCGGTA
BoDV_R	ATCATTCGATAGCTGCTCCCTTC
BoDV_probe	FAM-AGAACCCCTCCATGATCTCAGACCCAGA-TAMRA
